# OP74 Accelerated Approval Of Drugs In The USA: A Comparative Analysis Of United States Food And Drug Administration And Brazilian Regulatory Decisions

**DOI:** 10.1017/S0266462325101293

**Published:** 2025-12-29

**Authors:** Marcus Borin, Mariana Michel Barbosa, Carina Rejane Martins, Daniel Pitchon dos Reis, Geraldo José Coelho Ribeiro, Júlia Teixeira Tupinambás, Karina de Castro Zocrato, Lélia Maria de Almeida Carvalho, Marcela Pinto de Freitas, Maria da Glória Cruvinel Horta, Mariza Cristina Torres Talim, Sergio Adriano Loureiro Bersan, Silvana Marcia Bruschi Kelles

## Abstract

**Introduction:**

The United States Food and Drug administration (FDA) Accelerated Approval (AA) pathway facilitates early access to therapies for severe conditions based on surrogate endpoints, requiring confirmatory trials to validate benefits. This study compared FDA AA outcomes with Brazilian regulatory decisions, highlighting alignment and discrepancies, and their implications for patient safety and healthcare systems, focusing on post-market surveillance and evidence-based decision-making.

**Methods:**

Data on drugs on the FDA AA pathway were retrieved from the FDA’s publicly available repository as of 7 November 2024. Each drug was categorized by approval status: ongoing, verified clinical benefit, withdrawn, and other. Drugs were further analyzed for approval, coverage, and reimbursement decisions in Brazil using data from the Brazilian Health Regulatory Agency (ANVISA), the National Committee for Health Technology Incorporation (CONITEC), and the National Supplementary Health Agency (ANS). The analysis focused on discrepancies between FDA and ANVISA decisions and the implications for Brazilian healthcare systems.

**Results:**

A total of 336 drugs were analyzed: 53 percent achieved confirmed clinical benefit, 12.8 percent were withdrawn due to safety or efficacy concerns, and 30.7 percent are still undergoing confirmatory trials. The average time from AA to withdrawal was 7.35 years, highlighting extended patient exposure to unverified therapies. Of the 43 drugs withdrawn by the FDA, 12 retained active label indications by ANVISA. This regulatory lag impacts the Brazilian National and Supplementary Health Systems through potential inefficiencies in resource allocation.

**Conclusions:**

Discrepancies between FDA and ANVISA decisions underscore the need for stronger international collaboration on post-market surveillance and harmonized criteria for drug approvals. For Brazil, addressing these gaps could enhance patient safety, optimize healthcare resources, and align regulatory practices to ensure timely, evidence-based decision-making.

